# Supplementary material for: Flexible RSV Prefusogenic Fusion Glycoprotein Exposes Multiple Neutralizing Epitopes that May Collectively Contribute to Protective Immunity
Source: Vaccines (Basel). 2020 Oct 14;8(4):607. doi: 10.3390/vaccines8040607 (PMC7711572; doi:10.3390/vaccines8040607)
Supplement: Supplementary file 1 [file vaccines-08-00607-s001.pdf]

Flexible RSV prefusogenic fusion glycoprotein exposes multiple neutralizing epitopes that may collectively contribute to protective immunity

Nita Patel[1], Jing-Hui Tian[1], Rhonda Flores[1], Kelsey Jacobson[1], Michelle Walker[1], Alyse Portnoff[1], Mimi Gueber-Xabier[1], Michael J. Massare[1], Greg Glenn[1], Larry Ellingsworth[1], and Gale Smith[1]*

[1]Novavax, Inc. 21 Firstfield Road, Gaithersburg, MD, 20878, USA. npatel@novavax.com (N.P.), jhtian@Novavax.com (J.H.T.), rflores@Novavaax.com (R.F.), Kjacobson@Novavax.com (K.J.), mwalker@Novavax.com (M.W.), aportnoff@Novavax.com (A.P), mguebre-xabier@Novavax.com (M.G.X.), MMassare@Novavax.com (M.J.M.), gglenn@Novavax.com (G.G.), lellingsworth@gmail.com (L.E.), GSmith@Novavax.com (G.S.)

*Correspondence: GSmith@Novavax.com (G.S.)

Revised: date; Accepted: date; Published: date

Supplementary Materials: The following are available online at www.mdpi.com/xxx/sl

Appendix A

***Method:** Competition of RSV F Site-specific mAb with Polyclonal Antibodies to RSV F Proteins by Bio-layer Interferometry (BLI)*

Competitive binding of antigenic site-specific mAb with serum antibodies from immunized cotton rats was determined by BLI using an Octet OK384 instrument (Pall FortéBio, Menlo Park, CA, USA). Amine-reactive biosensor tips (Ar2G) were activated with 20 nM N-(3-dimethylaminopropyl)-N′-ethylcarbodiimide (EDC) and 10 mM N-hydroxy succinimide ester (NHS) buffer for 300 s. 100 nM prefusion F(BV2129) was coupled to the amine-reactive tips in 10 mM acetate buffer (pH 6.0) for 600 s using the manufacturer’s protocol. Unreacted amine was quenched with 1M ethanolamine followed by washing to baseline for 300 s. Prefusion F (BV2129) coupled biosensor tips were allowed to associate with serially diluted serum antibodies for 600 s. Washed biosensor tips were then exposed for 400 s with competing mAbs D25 (site Ø), hRSV90 (site V), palivizumab (site II), R1.42 (site IV), or R7.10 (p27). Competition between serum antibodies and the RSV F mAbs was analyzed with OctetHT10.0 software. Competing antibody equivalent (CAE) levels (μg mL^{-1}) were calculated based up on the percent competition, the concentration of competing antibody, and the serum dilution.

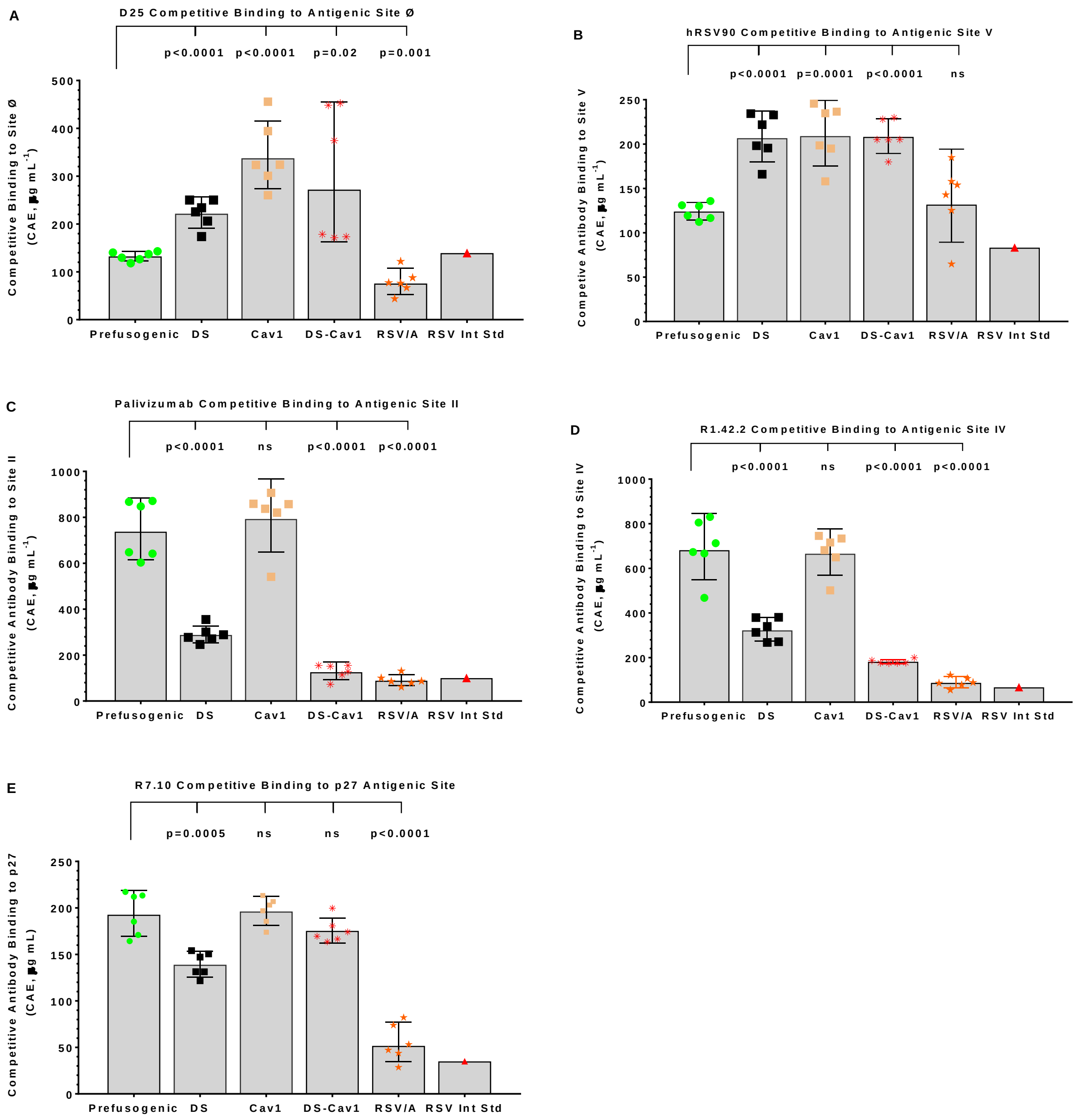

Figure S1. Competitive binding of RSV F site-specific monoclonal antibodies with immune serum determined by bio-layer interferometry (BLI) in cotton rats. Amine-reactive biosensor tips (ArG) were coupled to prefusion F (BV2129). Serum from cotton rats immunized with RSV F proteins was allowed to associate with biosensor tips for 600 s. Tips were washed and exposed for 400 s with site-specific competing mAbs. (**A**) D25 (site Ø). (**B**) hRSV90 (site V). (**C**) Palivizumab (site II). (**D**) R1.42 (site IV). (**E**) R7.10 (p27). Competition between serum antibodies and RSV F mAb was analyzed to OctetHT10.0 software. Horizontal bars indicate the group (N = 6 per group) GMT and the error bars indicate the 95% CI. Statistical significance between the RSV F prefusogenic vaccine group (BV1184) and paired immunized groups is indicated. Not significant (ns).
